# Knowledge, attitudes and practices towards COVID-19: Community survey in southern Ethiopia

**DOI:** 10.1371/journal.pone.0288430

**Published:** 2023-08-03

**Authors:** Misganu Endriyas, Endashaw Shibru, Mamush Hussen, Mintesinot Melka, Fiseha Lemango, Seyife Kibru, Degu Taye, Alelign Tadele

**Affiliations:** 1 SNNPR Public Health Institute, SNNPR Health Bureau, Hawassa, Sidama, Ethiopia; 2 Head Office, SNNPR Health Bureau, Hawassa, Sidama, Ethiopia; 3 School of Medicine and Health Sciences, Hawassa University, Hawassa, Sidama, Ethiopia; 4 Multisectoral HIV/AIDS Response Coordination, Sidama Regional Health Bureau, Hawassa, Sidama, Ethiopia; 5 Department of Laboratory, Hawassa Health Science College, Hawassa, Sidama, Ethiopia; University of Gondar College of Medicine and Health Sciences, ETHIOPIA

## Abstract

**Background:**

Being well informed about the pandemic and how the virus spreads help to prevent and control the pandemic. Health authorities should monitor community practice to prevent the pandemic to identify gaps and minimize risks. This study was, therefore, designed to assess community knowledge, attitude and practices (KAP) related to COVID-19 prevention and its associated factors in urban settings.

**Methods:**

Community based cross-sectional study was conducted in southern Ethiopia. Twelve towns with high population density and mobility and 1162 participants were included in the study using multi-stage sampling. Semi-structured questionnaire was used to collect data. Mask use was assessed by observation while social distancing and handwashing were assessed by interview. Good practice was defined as wearing face mask, keeping social distance and handwashing. Data was collected by health professionals who have BSc and above (in nursing and public health) and analyzed using SPSS version 25. Descriptive statistics and binary logistic regression at 95% confidence level were performed.

**Results:**

From 1162 respondents, about three fifths, 714 (61.4%), were females and 829 (64.2%) were married. The mean knowledge score was 69.7 (SD±17.87) while mean score for attitude was 80.6 (SD±6.29). Only about one third, 380 (32.7%), had good practice. Occupation, age and overall knowledge about COVID-19 and its prevention were associated with good COVID-19 prevention measures.

**Conclusion:**

Knowledge and attitude related to COVID-19 prevention and control were moderate while practice was low. The risk communication strategy should be strengthened using precautionary advocacy.

## Background

The Coronavirus disease 2019 (COVID-19) is highly infectious viral disease that can cause mild to serious respiratory illness [1–3]. Although efforts have been made to control COVID-19, the pandemic has been causing huge impact on social, economic and healthcare systems being cause for millions of cases and deaths globally [4, 5]. As of Nov 30, 2020, there were 64,653,909 cases and 1,604,896 deaths globally. The same report showed that 109,534 cases and 1,700 deaths were registered in Ethiopia [6].

In developing countries especially in in urban settings, the COVID-19 prevention has been challenging because of dense population, small informal dwellings, lack of access to clean water, multi-generational households with shared sanitation facilities, high level of social mixing, and transient residents [7, 8].

According to the World Health Organization, being well informed about the disease and how the virus spreads helps to prevent and control the pandemic [3]. Since early introduction of the disease to Ethiopia and study region, a lot has been done to aware community and adhere prevention measures. Some of these activities include supplies like face mask and sanitation materials, and health education through mass media (radio, TV, social media etc.). Previously, to assist these responses, a survey was done to assess knowledge, attitude and practice related to COVID-19 prevention activities and its associated factors in selected zonal towns in Southern, Nations, Nationalities and Peoples’ Region (SNNPR), Ethiopia [9]. Then, awareness creation and risk communication were continued to strengthen community responses focusing on identified gaps. Later, there was a need of information on changes in knowledge, attitude and practice regarding COVID-19 and its prevention. So, this survey was designed to assess knowledge, attitude and practice (KAP) related to COVID-19 prevention activities and its associated factors in urban settings of southern Ethiopia.

## Methods

### Study design and setting

This community-based cross-sectional study was carried out from October 15 to November 05, 2020 in selected 12 towns of SNNPR. After previous survey, the region was administratively divided in to two regions, Sidama and SNNPR. The new SNNPR was the third largest administrative region of Ethiopia representing about 15% of the country’s population comprising 16 zones and 7 special woredas (districts). Currently, after this study, the region was redivided in to two regions: Southwest Ethiopia and SNNPR.

### Sample size and sampling

Sample size was estimated using a single population proportion formula at 95% confidence level assuming proportion of population with good practice to be 48.9% from previous survey [9] and considering 5% margin of error, design effect of three and 10% non-response rate and the final sample size was 1258. The calculated sample size was allocated to ten zonal towns based on the size of population. Twelve towns (11 zonal capital towns and 1 non-zonal town) were purposely selected considering potential risk of COVID-19, crowdedness and high mobility.

**Fig 1:**
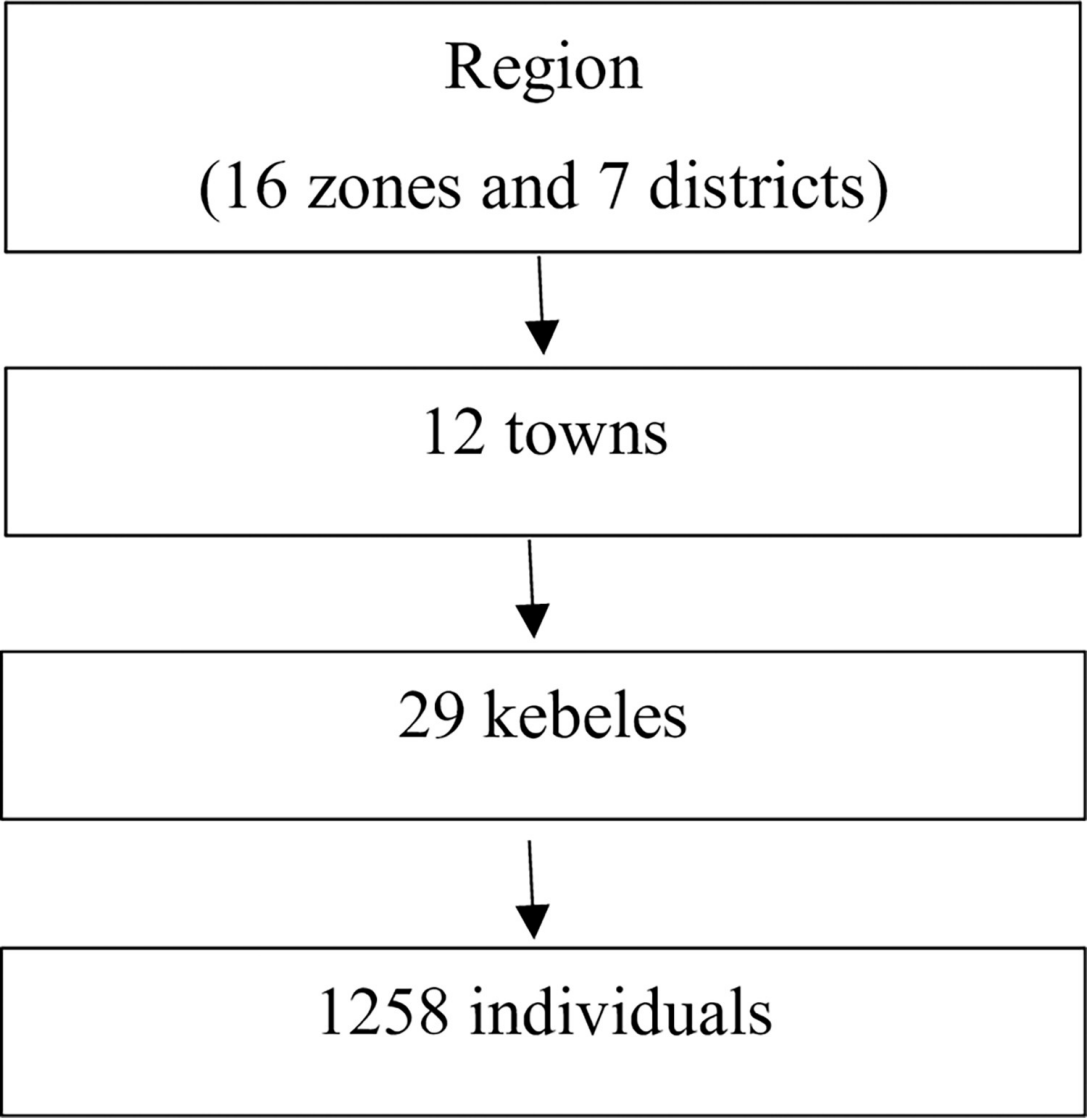
Sampling procedure.

We used similar methods of sampling (Fig 1), data collection tools and data management techniques that were applied during previous survey [9]. Multistage sampling was used to select study participants. Two to four Kebeles, smallest administrative structures, per town were selected based on sample size. For sample size less than 100, two kebeles were selected. For towns with sample size of 101 to 200, three kebeles were selected and for those above 200, four kebeles were selected. In this way, four kebeles were selected from one town, three kebeles selected from three towns and two kebeles selected from the rest. Data collectors went to the center of selected kebeles and selected first house by pining pen. The next household was then selected from next adjacent block in parallel position as pinned pen and continued until sample size was fulfilled. In previous survey, participants staying at house and walking outside were included while this survey included household survey only.

### Data collection methods

Few questions were added to previous questions considering responses from previous survey. Twelve questions were used assess knowledge and questions with multiple responses were operationalized as presented in Table 2. Thirteen different attitude statements including seriousness of disease, being at risk, possibility of prevention and benefits of staying at health facilities and politicizing COVID-19 (considering as propaganda) were given to respondents and their agreement to statements was measured using Likert’s scale out of five, from strongly disagree to strongly agree. Although respondents reported different measures to prevent the disease, we considered social distancing, wearing face mask and handwashing as good practice. Social distancing and handwashing were measured by self-report while wearing face mask was measured by observation during interview.

Nine data collectors who had BSc degree and above (in nursing and public health) and have experience in data collection, including previous survey, conducted interviews keeping COVID-19 prevention measures. Online monitoring and field supervision were done daily to maintain data quality.

### Data management

Excel file downloaded from SurveyCTO server was imported to SPSS version 25 for data management (S1 Data). Variables were transformed to best fit for analysis. Responses in “others” were reviewed and categorized based on similarity of contents. Descriptive statistics and binary logistic regression were performed to describe and predict factors associated with good practice. Each of knowledge questions were recoded to 1 (correct) or 0 (incorrect) based on specific criterium. Values of responses to negative attitude statements were reversely recoded to get value of positive statements. For example, strong disagreement or 1 (strongly disagree) for negative attitude was recoded to 5 (strongly agree) to indicate correct response. Both knowledge and attitude scores were summed and quantile was used to summarize categories. Variables with p-value of less than 0.05 during multivariable binary logistic regression were considered as factors associated with good practice and were reported with adjusted odds ratio (AOR) at 95%CI.

### Ethical considerations

Ethical clearance was obtained from the ethical review committee of the regional health bureau (Reference number: የወ6-19/6193). Informed verbal consent was approved and used because of its popularity in study settings and to minimize contamination during the pandemic. Questionnaire on SurveyCTO was set only to open questions if participant agreed to continue after data collectors read the information (purpose/objective, right to participate or not, privacy etc) on information sheet, and SurveyCTO closed survey form if consent was disagreed. No personal identifier was collected, and all collected data are kept confidential.

## Results

Overall, 1162 respondents were included in the study, with response rate of 92.4%; non-response mainly to social distancing. About three fifths, 714 (61.4%), of respondents were females and 829 (64.2%) were married. About one third, 398 (34.3%), of respondents were Orthodox followers and about two thirds, 783 (67.4%), were from households with family size of five or less (Table 1).

**Table 1:** Socio-demographic characteristics of respondents, SNNPR.

Variables	Category	Frequency	Percent
Sex	Male	448	38.6
	Female	714	61.4
Age	< = 25	311	26.8
	26–30	215	18.5
	31–35	159	13.7
	36–40	134	11.5
	41–45	96	8.3
	46+	174	15.0
	I don’t know	73	6.3
Marital status	Single	284	24.4
	Married	829	71.3
	Widowed/divorced	49	4.2
Educational status	No formal education	101	8.7
	Primary (1–8)	257	22.1
	Secondary (9–12)	343	29.5
	Certificate and above	461	39.7
Occupation	Student	192	16.5
	Farmer	21	1.8
	Merchant	287	24.7
	Employee	282	24.3
	Housewife	267	23.0
	Daily labor	38	3.3
	Pensioner	23	2.0
	Others*	52	4.5
Family size	≤ 5	783	67.4
	≥ 6	379	32.6
Religion	Orthodox	398	34.3
	Protestant	532	45.8
	Muslim	212	18.2
	Catholic/ Adventist/Pagan	22	1.7

*- Evangelist, home servant, motorist, carpenter, footballer, mechanic, weaver and jobless

### Knowledge about and attitude towards COVID-19

**Fig 2:**
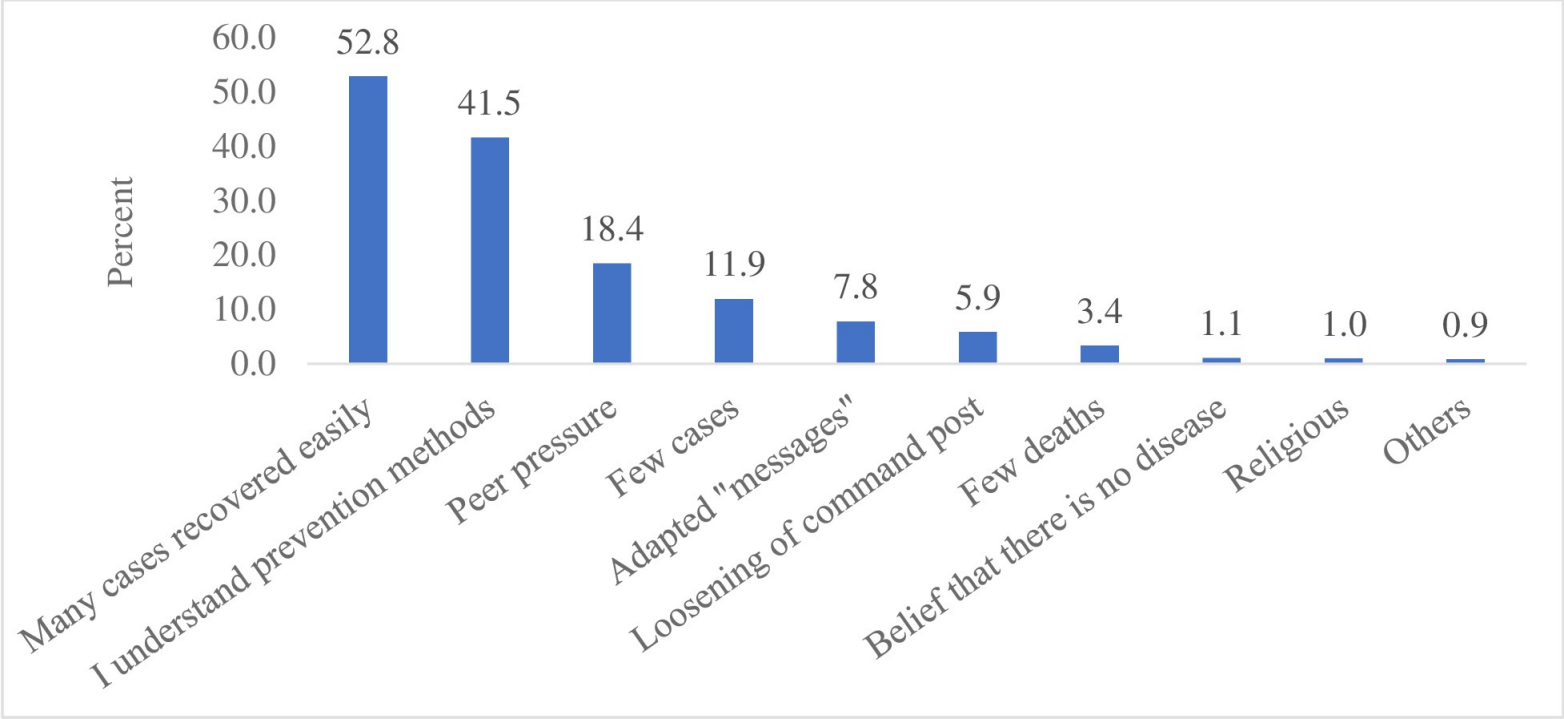
Reasons for decreasing fear towards COVID-19.

The knowledge score ranged from zero to 100% with mean score of 69.7% (SD±17.87). Highest scores were recorded on questions regarding transmissibility of the disease and isolation of suspects while lowest scores were recorded on questions about sign and symptoms and port of entry when three items were considered for each question (Table 2). For thirteen attitude questions (Table 3), minimum expected score was 13 and maximum was 65. The respondents mean score for attitude was 80.6% (SD±6.29), ranging from 52.3 to 100%. In addition to attitude questions, we asked respondents to classify their fear towards the disease as “the same”, “decreased” or “increased” considering current and early times. Majority, 922 (79.4%), reported that their fear towards the disease was decreased while only 112 (9.6%) reported it was increased mainly due to high number of cases. Reasons for decrease in fear were presented in Fig 2. The leading reason for decrease in fear was that many cases recovered easily (52.8%), followed by understanding prevention methods (41.5%).

**Table 2:** Knowledge of COVID-19 and its prevention and control in urban settings, SNNPR.

Knowledge questions	Frequency	Percent
Know signs and symptoms (fever, cough AND difficulty in breathing)	462	39.8
Know confirmation (lab test)	530	49.9
Know what to do if there is suspect (reporting to health facility)	1038	89.3
Know what to do if develop sign and symptoms (reporting to health facility)	1006	86.6
Know that close contacts should be isolated (true)	1093	94.1
Know that there is no drug to cure	988	85.0
Know that COVID-19 is transmissible	1147	98.7
Know route of entry (mouth, nose AND eye)	391	33.6
Know prevention (social distancing, clean hands AND wearing mask)	720	62.0
Know most at-risk groups (old age and people with underlying sickness)	528	45.4
Know that not all cases show symptoms	796	68.5
Know that asymptomatic cases can transmit COVID-19	967	83.2

**Table 3:** Attitude towards COVID-19 and its prevention and control in urban settings, SNNPR.

Statements	Strongly disagree	Disagree	Neutral	Agree	Strongly agree
Covid is dangerous disease	3 (0.3)	41 (3.5)	24 (2.1)	624 (53.7)	470 (40.4)
I feel am vulnerable to contracting COVID-19.	14 (1.2)	111 (9.6)	59 (5.1)	766 (65.9)	212 (18.2)
I feel my family members are vulnerable to contracting COVID-19.	12 (1.0)	81 (7.0)	69 (5.9)	771 (66.4)	229 (19.7)
I fear to go to crowded places.	3 (0.3)	106 (9.1)	32 (2.8)	826 (71.1)	195 (16.8)
If I practice prevention methods, I can prevent COVID-19.	0 (0.0)	12 (1.0)	32 (2.8)	867 (74.6)	251 (21.6)
If others people practice COVID-19 prevention methods, they can prevent COVID-19.	2 (0.2)	19 (1.6)	29 (2.5)	870 (74.9)	242 (20.8)
COVID-19 cases cannot be cured with medical follow up.	164 (14.1)	770 (66.3)	62 (5.3)	130 (11.2)	36 (3.1)
For people with COVID-19 symptoms, going to health facility has no benefit.	186 (16.0)	802 (69.0)	31 (2.7)	95 (8.2)	48 (4.1)
We can prevent COVID-19 by washing hands with soap.	5 (0.4)	47 (4.0)	28 (2.4)	900 (77.5)	182 (15.7)
COVID-19 patients should stay in treatment centers.	2 (0.2)	37 (3.2)	21 (1.8)	898 (77.3)	204 (17.6)
I will be in treatment center if I contract COVID-19.	0 (0.0)	42 (3.6)	30 (2.6)	866 (74.5)	224 (19.3)
Youths and children should not take care of COVID-19 because they are not at high risk.	439 (37.8)	591 (50.9)	102 (8.8)	24 (2.1)	6 (0.5)
COVID-19 is political than a serious disease.	226 (19.4)	654 (56.3)	207 (17.8)	56 (4.8)	19 (1.6)

### Practice regarding COVID-19 prevention

Respondents answered multiple methods as part of COVID-19 prevention. The most common method was handwashing (89.2%), followed by wearing face mask (55.1%) (Table 4). Considering social distancing, wearing face mask and handwashing as good practice, 380 (32.7%) were practicing it in a proper way.

**Table 4:** COVID-19 prevention methods practiced by respondents in urban settings, SNNPR.

Knowledge questions	Frequency	Percent
Social distancing (staying at home)	627	54.0
Handwashing	1037	89.2
Avoid touching eye and nose	296	25.5
Wear face mask	640	55.1
Drink hot drinks	271	23.3
Keep personal hygiene and environmental sanitation	162	13.9
Use traditional medicines	124	10.7
Do physical exercise	53	4.6
Others (stopped shaking hands, pray, drink alcohol, cough or sneeze into your elbow, do nothing)	39	3.3

### Factors associated with COVID-19 prevention practice

Educational level, occupation, age, level of knowledge and attitude were associated at bivariate level and considered for multivariable regression. After running multivariable binary logistic regression, occupation, age and overall knowledge about COVID-19 and its prevention were associated with good COVID-19 prevention measures (Table 5).

**Table 5:** Bivariable and multivariable binary logistic regression on factors associated with good COVID-19 prevention, SNNPR.

Variable	Category	Practice COVID-19 prevention		COR 95%CI	AOR 95%CI
		No	Yes		
Sex	Male	304	144	1	
	Female	478	236	1.04 [0.81–1.34]	
Marital status	Single	193	91	1	
	Married	555	274	1.05 [0.78–1.40]	
	Widowed/divorced	34	15	0.94 [0.48–1.80]	
Educational status	No formal education	72	29	1	
	Primary (1–8)	188	69	0.91 [0.54–1.52]	0.94 [0.53–1.68]
	Secondary (9–12)	239	104	1.08 [0.66–1.76]	1.07 [0.59–1.92]
	Certificate and above	283	178	1.56 [0.97–2.50]	1.00 [0.54–1.85]
Occupation	Student	130	62	1	
	Farmer	16	5	0.66 [0.23–1.87]	0.91 [0.28–3.00]
	Merchant	211	76	0.76 [0.51–1.13]	0.93 [0.56–1.52]
	Employee	166	116	1.46 [0.99–2.15]	1.83 [1.07–3.10]
	Housewife	182	85	0.98 [0.66–1.46]	1.51 [0.90–2.51]
	Daily labor	32	6	0.39 [0.16–0.99]	0.59 [0.22–1.61]
	Pensioner	11	12	2.89 [0.96–5.47]	3.57 [1.25–10.24]
	Others*	34	18	1.11 [0.58–2.11]	1.59 [0.78–3.22]
Age category	< = 25	211	100	1	
	26–30	143	72	1.06 [0.73–1.54]	0.79 [0.51–1.23]
	31–35	121	38	0.66 [0.43–1.02]	0.49 [0.29–0.83]
	36–40	95	39	0.87 [0.56–1.35]	0.57 [0.33–0.97]
	41–45	66	30	0.96 [0.58–1.57]	0.78 [0.44–1.41]
	46+	119	55	0.97 [0.66–1.45]	0.70 [0.41–1.19]
	I don’t know	27	46	3.59 [2.11–6.12]	3.17 [1.74–5.77]
Family size	≤ 5	520	263	1	
	≥ 6	262	117	0.88 [0.68–1.15]	
Overall knowledge	Poor	321	78	1	
	Medium	262	141	2.22 [1.61–3.05]	2.14 [1.51–3.03]
	Good	199	161	3.33 [2.41–4.60]	3.38 [2.36–4.84]
Overall attitude	Negative attitude	233	91	1	
	Neutral	379	166	1.12 [0.83–1.52]	0.97 [0.70–1.34]
	Positive attitude	170	123	1.85 [1.33–2.59]	1.42 [0.99–2.05]

*- Evangelist, home servant, motorist, carpenter, footballer, mechanic, weaver and jobless

Employees and pensioners were 1.83 and 3.57 times more likely to practice COVID-19 prevention as compared to students; AOR at 95%CI: 1.83 (1.07–3.10) and 3.57 (1.25–10.24) respectively.

Age categories from 31–35 and 36–40 were 51% and 43% less likely to practice COVID-19 prevention while those who do not know their age were 3.17 times more likely to practice COVID-19 prevention as compared to age groups less or equal to 25 years; AOR at 95%CI: 0.49 (0.29–0.83), 0.57 (0.33–0.97) and 3.17 (1.74–5.77) respectively.

Respondents who had medium and good level knowledge scores were 2.14 and 3.38 times more likely to practice COVID-19 prevention measures as compared to respondents with poor knowledge scores; AOR at 95%CI: 2.14 (1.51–3.03) and 3.38 (2.36–4.84) respectively.

## Discussion

This study was done with the objective of assessing KAP related to COVID-19 and factors associated with COVID-19 prevention practices. Major finding included that the level of knowledge was improved while fear towards the disease and good prevention practices were decreased as compared to results of study done during early introduction of the pandemic to the region. Only about one third of participants were practicing COVID-19 prevention measures in a good way, and it was associated with occupation, age and overall knowledge about COVID-19 and its prevention.

The adherence to public health recommendations to control COVID-19 spread is influenced by KAP of the public [10]. KAP surveys can help in collecting information on the knowledge, opinion, attitude, and behavioral practices and can be used as evaluation of interventions [11]. In Ethiopia, number of studies have been done on KAP of COVID-19 but reported varying level of KAP [12–19]. So, this study reports the KAP in southern Ethiopia by comparing with previous study done in similar setting.

The overall knowledge score increased from 52.3% in previous survey [9] to 69.7% (SD±17.87) in current survey. This improvement in knowledge was highly appreciated, but notable gaps had been recorded in understanding key signs and symptoms, the most at-risk groups and route of infection as indicated in Table 2. Since health education programs aimed at improving the public’s understanding of COVID-19 are important to maintain appropriate practices [20], the existing risk communication should be strengthened.

The mean attitude score was almost equal with previous survey, 80.8% [9] vs 80.6%. This could be due to addition of few questions to attitude questions. However, the majority (79.4%) of participants reported that their fear towards the disease was decreased mainly due to the reason that many cases recovered easily (52.8%), followed by understanding prevention methods (41.5%).

The good COVID-19 prevention practice, however, decreased from previous survey. In previous survey, the good practice defined as practicing social distancing, using face mask and handwashing was 48.9% while it was only 32.7% in current survey using similar method of measurement. During previous survey, people tried to practice even with poor knowledge. A study done in Pakistan also reported high level of good practices (95.0%) with lower level of knowledge (67.4%) [21]. A study in Ethiopia also reported higher level of good practice (59.8%) while there was low level of good knowledge (37.2%) [19]. The decrease in good practice during current survey could be due to loosening of restrictions of state of emergency and decrease in fear towards the disease.

Employees were 1.83 times more likely to practice COVID-19 prevention as compared to students; AOR at 95%CI: 1.83 (1.07–3.10). In addition, respondents who had medium and good level knowledge scores were 2.14 times and 3.38 times more likely to practice COVID-19 prevention measures as compared to respondents with poor knowledge scores; AOR at 95%CI: 2.14 (1.51–3.03) and 3.38 (2.36–4.84) respectively. The level of education and level of knowledge have been positively reported as significant factor for good COVID-19 prevention practices in studies conducted in Sidama, Tigrai and Southwest regions of Ethiopia [22–24] and previous survey [9], and also study done in Palestine [20]. Since employees are educated, they are supposed to have good knowledge and that could have influenced their practice.

Those participants who did not know their age were 3.17 times more likely to practice COVID-19 prevention as compared to age groups less or equal to 25 years, AOR at 95%CI: 3.17 (1.74–5.77). Although participants who did not know their age can fall in any age categories, these groups are usually people in old ages. Pensioners were also 3.57 times more likely to practice COVID-19 prevention as compared to students, AOR at 95%CI: 3.57 (1.25–10.24). Since pensioners are also usually aged people, this could be due to seriousness of illness among old ages and intensive health educations targeted towards old ages. Furthermore, age categories from 31–35 and 36–40 were 51% and 43% less likely to practice COVID-19 prevention as compared to age groups less or equal to 25 years, AOR at 95%CI: 0.49 (0.29–0.83) and 0.57 (0.33–0.97) respectively. This might be due to the reason that the age group between 31 and 40 years are highly productive and their engagements in different activities that challenge them to properly practice COVID-19 prevention measures like social distancing.

Risk communication activities can modify the successful implementation of public health interventions [25]. Risk communication is one of key sections in emergency operations center (EOC) and has been transmitting messages through different medias to improve implementation of COVID-19 prevention measures. In the study settings, fortunately, the pandemic was not serious as expected and also as the message transmitted. This resulted in fast resistance by the community to fear arousal messages transmitted by stakeholders. For example, majority (79.4%) of participants reported that their fear towards the disease was decreased due to the reason (mainly) that many cases recovered easily (52.8%). The risk communication strategy commonly implemented at the time was crisis communication (fear arousal). Since failure to communicate the right message effectively can result in loss of trust, economy and lives [26], we recommend the shift of approach from crisis communication to precautionary advocacy, which is appropriate in the cases of alerting insufficiently concerned people to serious hazards [27, 28].

Although we covered major towns in the region, the study was limited in addressing smaller towns and rural areas that account for majority of regional population. In addition, some of reported practices like social distancing and handwashing might have been knowledge and may not be actual practice of respondents due to social desirability bias.

## Conclusion

Knowledge and attitude related to COVID-19 prevention and control were moderate while COVID-19 prevention practice was low. Occupation, age and overall knowledge about COVID-19 and its prevention were associated with practicing COVID-19 prevention measures. The risk communication should be strengthened and its strategy should be shifted from crisis communication (fear arousal) to precautionary advocacy.

## Supporting information

S1 Data Data in SPSS sav file type. (SAV)
